# Neurological Manifestations of Long COVID: Current Evidence, Emerging Concepts, and Future Perspectives

**DOI:** 10.3390/jcm15187163

**Published:** 2026-09-15

**Authors:** Marina Bralic, Frederic-Ivan Silconi, Vladimira Vuletic, Slavica Kovacic

**Affiliations:** 1 Department of Neurology, Faculty of Medicine, University of Rijeka, Braće Branchetta 20, 51000 Rijeka, Croatia; vladimira.vuletic@uniri.hr; 2 Department of Neurology, University Hospital Centre Rijeka, 51000 Rijeka, Croatia; 3 Department of Neurology, General Hospital Pula, 52100 Pula, Croatia; fsilconi@hotmail.com; 4 Department of Radiology, Faculty of Medicine, University of Rijeka, 51000 Rijeka, Croatia; slavica.kovacic@uniri.hr; 5 Department of Radiology, University Hospital Centre Rijeka, 51000 Rijeka, Croatia

**Keywords:** COVID-19, Long COVID, nervous system diseases, biomarkers

## Abstract

Six years after the onset of the COVID-19 pandemic, many individuals continue to experience persistent neurological symptoms that extend beyond the acute phase of infection, affecting daily life, work, and overall functioning, thereby representing a substantial long-term health burden. Cognitive impairment, fatigue, headache, and autonomic dysfunction are among the most frequently reported neurological features, often persisting despite the absence of clear diagnostic findings. Although Long COVID is recognized as a distinct clinical entity, the underlying pathophysiological mechanisms remain incompletely understood. This narrative review provides a critical synthesis of current literature regarding the clinical presentations, potential mechanisms, and biomarkers associated with neurological involvement in Long COVID. Reflecting the high heterogeneity of the available evidence, it is important to note that no neurological biomarker or disease-modifying therapy has yet been clinically validated. Ultimately, characterizing these pathways may help define disease heterogeneity, support patient stratification, and guide future personalized therapeutic approaches.

## 1. Introduction

Six years after the emergence of severe acute respiratory syndrome coronavirus 2 (SARS-CoV-2), a substantial proportion of individuals continue to report symptoms that persist beyond the acute phase of infection [1]. The term “Long COVID” has become widely used to describe this condition [1,2]. It encompasses a broad range of persistent, recurrent, or newly emerging symptoms affecting multiple organ systems, which may occur irrespective of the severity of the initial infection and may last for weeks, months, or longer [2,3,4]. Fatigue, shortness of breath, and cognitive difficulties are among the most frequently reported complaints, although more than 200 symptoms have been described in association with Long COVID [2]. Long COVID has consequently become an important clinical and public health concern [2,3,4].

Neurological manifestations form an important part of this spectrum. Patients may experience cognitive difficulties, memory impairment, headache, paresthesias and other sensory disturbances, balance problems, fatigue, and autonomic symptoms, with some of these manifestations substantially affecting everyday functioning [5,6].

Several reviews and meta-analyses have examined neurological and neuropsychiatric manifestations following SARS-CoV-2 infection. Early reviews and meta-analyses described a broad range of persistent symptoms, including fatigue, “brain fog,” headache, cognitive and sleep disturbances, sensory symptoms, myalgia, and dysautonomia [7,8]. Subsequent systematic reviews and meta-analyses provided further estimates of the frequency and persistence of neurological manifestations, particularly fatigue, cognitive impairment, headache, and difficulties with memory and attention [6,9,10]. More recent reviews have expanded the focus to neurological phenotypes, potential mechanisms, and diagnostic and therapeutic considerations, while also emphasizing the continuing uncertainty surrounding the biological basis of these symptoms [6,11]. A systematic review examining central nervous system manifestations persisting for 12 months or longer further demonstrated the substantial neurological burden associated with Long COVID [12]. Similarly, a large systematic review and meta-analysis published in 2025, including more than 4 million participants, provided additional evidence of persistent neurological and cognitive consequences following SARS-CoV-2 infection [13].

Alongside the clinical evidence, research into the biological basis of neurological Long COVID has expanded considerably. Several mechanisms have been proposed, including persistent immune activation and neuroinflammation, endothelial and blood–brain barrier dysfunction, autonomic disturbances, altered serotonin and tryptophan metabolism, mitochondrial dysfunction, and other vascular and metabolic abnormalities [3,4,14,15,16,17]. Biomarker research has developed in parallel, with studies investigating indicators of neuronal and glial injury, inflammatory and immune activation, endothelial dysfunction, and autoimmunity [18,19,20,21]. Nevertheless, findings have been inconsistent across studies, and no individual biomarker has yet demonstrated sufficient specificity, reproducibility, and clinical validity for routine use in neurological Long COVID [20,21].

Despite the rapid expansion of this literature, important gaps remain. Clinical phenotypes, proposed mechanisms, and biomarker findings have often been investigated separately, making it difficult to determine how they relate to one another [11,19,20,21]. Differences in Long COVID definitions, patient populations, follow-up periods, clinical outcome measures, and laboratory methodologies further limit direct comparison between studies [2,3,4,5]. At the same time, the growing body of biomarker research has not yet been fully integrated with the clinical and pathophysiological heterogeneity of neurological Long COVID.

This narrative review provides an updated and critical synthesis of neurological Long COVID, bringing together its clinical manifestations, underlying mechanisms, emerging biomarkers, diagnostic considerations, treatment approaches, and recovery trajectories. Particular attention is given to the heterogeneity of neurological presentations and to the distinction between established neurological disease, consequences of severe systemic illness, and symptoms without a demonstrable structural correlate. The available mechanistic and biomarker evidence is critically examined, including areas of uncertainty and conflicting findings. The review also considers priorities for future research, including biomarker validation, patient stratification, and individualized treatment.

## 2. Materials and Methods

### 2.1. Literature Search Strategy

This study was designed as a narrative review focusing on the neurological manifestations, potential underlying mechanisms, and biomarkers associated with Long COVID. A comprehensive literature search was conducted primarily using PubMed, supplemented by Google Scholar, with the final search conducted on 15 July 2026.

The initial search combined MeSH terms and free-text keywords related to Long COVID and neurological manifestations. Targeted searches were subsequently conducted for specific neurological manifestations, pathophysiological mechanisms, therapeutic approaches, and biomarkers. Additional relevant studies were identified by screening reference lists. The complete search strategies are provided in Supplementary Table S1.

A general Google search was used exclusively to identify official clinical guidelines, consensus statements, and institutional documents from established health organizations, such as World Health Organization (WHO) and Centers for Disease Control and Prevention (CDC).

Reference lists of all retrieved articles were manually screened to identify additional relevant studies. The complete search strings, database platforms, and detailed search strategies are provided in Supplementary Table S1 to ensure transparency and reproducibility.

### 2.2. Selection, Data Extraction, and Critical Synthesis

Publications were considered for inclusion when they addressed neurological manifestations of Long COVID or post-COVID-19 condition, including clinical manifestations, potential pathophysiological mechanisms, therapeutic approaches, or biomarkers associated with neurological involvement. Particular attention was given to studies addressing cognitive impairment, fatigue, headache, autonomic dysfunction, neuroinflammation, blood–brain barrier dysfunction, and neurological or circulating biomarkers potentially associated with Long COVID.

Original research studies, clinical and observational studies, longitudinal cohort studies, controlled studies, and relevant systematic or narrative reviews were considered according to their relevance to the specific topics addressed in the review. Studies focusing exclusively on acute COVID-19 without addressing persistent or post-acute neurological manifestations were not considered relevant to the scope of this review.

Relevant information was extracted from the identified literature on study characteristics, patient populations, neurological manifestations, proposed pathophysiological mechanisms, therapeutic approaches, and investigated biomarkers. Differences in study design, sample size, follow-up duration, diagnostic definitions, control groups, and methodological approaches were taken into account when interpreting the available evidence.

To ensure a transparent and critical synthesis, study findings were interpreted according to a predefined methodological hierarchy. The greatest weight was assigned to evidence derived from large-scale meta-analyses and prospective controlled cohort studies utilizing well-characterized populations and standardized, objective outcome measures. Intermediate weight was given to smaller longitudinal or cross-sectional observational studies with appropriate control groups. Preliminary biomarker findings, small case series, and expert consensus guidance based on low-certainty data were interpreted cautiously and considered exploratory rather than definitive.

The available evidence was synthesized qualitatively and organized thematically according to the major topics addressed in the review. Given the narrative design of the review and the considerable heterogeneity of the literature with respect to study populations, definitions of Long COVID, study designs, clinical assessments, and biomarker methodologies, no quantitative meta-analysis or formal risk-of-bias assessment was performed.

## 3. Long COVID Definition

Initially, there was no globally accepted definition of Long COVID. Persistent symptoms following the acute phase of SARS-CoV-2 infection have been described using various terms, including “post-COVID,” “Long COVID,” “post-COVID-19 condition,” and “post-acute sequelae of SARS-CoV-2 infection (PASC)” [1]. In October 2021, the World Health Organization (WHO), based on a Delphi consensus, defined post-COVID-19 condition as a condition occurring in individuals with probable or confirmed SARS-CoV-2 infection, usually 3 months from the onset of COVID-19, with symptoms lasting for at least 2 months and not explained by an alternative diagnosis. These manifestations may emerge following initial recovery, persist from the acute phase, or fluctuate and relapse over time [1].

In this review, we use the term Long COVID to denote persistent, recurrent, or newly emerging clinical manifestations occurring usually 3 months after SARS-CoV-2 infection, in the absence of an alternative diagnosis that better explains the presentation [1].

Although Long COVID is clinically and biologically heterogeneous and may persist for years in some individuals, the 3-month threshold is used here as an operational timeframe to distinguish it from acute and early post-acute COVID-19 [2,3].

However, the WHO definition provides a clinical framework for case identification rather than a definitive biological diagnosis, as no single validated biomarker or diagnostic test currently exists [1]. As documented by Yang et al., differences in case definitions and methods used to identify and measure Long COVID in real-world practice contribute to substantial heterogeneity across study populations, complicating comparisons between cohorts and limiting the reproducibility of findings [5]. Pending the discovery of specific biomarkers, phenotype-based characterization serves as a pragmatic interim approach to supplement the broad WHO criteria. Stratifying patients into predominant clinical domains, such as neurological/cognitive, autonomic, cardiopulmonary, fatigue/post-exertional symptom exacerbation (PESE), and multisystem presentations, complements case identification [22,23]. Information on symptom trajectory, functional impairment, severity of the initial infection, vaccination and reinfection history, and relevant comorbidities may further assist clinical assessment and comparison across cohorts. This type of stratification should be considered a pragmatic interim approach rather than a validated diagnostic classification [2,3,5].

## 4. Epidemiology

A global assessment of the burden of Long COVID remains challenging because reported prevalence estimates vary substantially across studies and should not be interpreted as directly comparable measures of a single, uniform outcome. This variability reflects differences in Long COVID definitions, study populations and denominators, disease severity, vaccination status, circulating SARS-CoV-2 variants, timing and duration of follow-up, the presence or absence of non-infected control groups, and methods used to ascertain symptoms [24,25]. Prevalence estimates are therefore best interpreted in the context of the underlying study design and population characteristics rather than as fixed estimates of disease frequency [24,25].

Long COVID has been reported across the full age spectrum, although its prevalence and clinical characteristics may vary substantially by age and study population [2,26]. The 10–20%, 10–30%, and 45% estimates refer to different populations, follow-up periods, case definitions, and thresholds, including the presence of “at least one persistent symptom.” Specifically, approximately 10–30% of non-hospitalized individuals in community cohorts have been reported to develop persistent symptoms, with substantially higher proportions observed in some hospitalized cohorts [2]. In contrast, global estimates provided by the World Health Organization (WHO) suggest that approximately 10–20% of people who have experienced COVID-19 may develop Long COVID, across the spectrum of initial disease severity [27]. Furthermore, a systematic review and meta-analysis including 194 studies and 735,006 participants reported an estimate of approximately 45%; however, this figure was based on COVID-19 survivors with at least one unresolved symptom at a mean follow-up of 126 days [28].

These apparently discrepant estimates are not necessarily contradictory, but largely reflect differences in case definitions, target populations, follow-up periods, symptom thresholds, and outcome ascertainment [24,25,28].

Case definition and outcome ascertainment are among the most important determinants of reported prevalence [24]. Earlier studies used heterogeneous approaches, ranging from the persistence of individual symptoms to predefined symptom duration or clinical criteria [5,23]. Studies relying on self-reported symptoms, questionnaires, or online surveys may produce different estimates from those using standardized clinical assessments, while self-selection and participation bias may further influence survey-based estimates [24,25,29]. These methodological differences are particularly relevant when comparing prevalence across studies or estimating the population-level burden of Long COVID [24,25].

Study population and disease severity represent additional major sources of heterogeneity. While hospitalized patients with severe acute illness typically exhibit an elevated risk of persistent symptoms compared to individuals with mild or asymptomatic community-managed infections, estimates derived from these inpatient cohorts cannot be directly generalized to the broader infected population [2,28,30]. The denominator is also critical, as prevalence calculated among all infected individuals, symptomatic individuals, hospitalized patients, or survivors represents different epidemiological measures [24,28]. The inclusion of appropriate non-infected control groups is similarly important, particularly for symptoms common in the general population, as it allows the excess burden associated with infection to be distinguished from background symptom prevalence [24].

Timing and duration of follow-up further influence reported prevalence. Long COVID symptoms may fluctuate, resolve, or emerge over time, and estimates obtained at three months cannot be assumed to be equivalent to those obtained at six or twelve months [23,28,30]. Differences in the timing of assessment relative to the acute infection may produce substantially different estimates even in otherwise comparable populations. Retrospective studies may also be affected by recall bias, particularly when symptoms are assessed many months after the initial infection [24,25].

The evolving epidemiological context of the pandemic further complicates comparisons across cohorts. Studies conducted during different periods included individuals exposed to different variants and levels of population immunity. Vaccination status, previous infection, variant exposure, and severity of acute illness may influence both the risk and clinical phenotype of Long COVID [25,31]. Changes in circulating variants, population immunity, and acute management limit direct comparisons between early- and later-pandemic cohorts [25,31].

Taken together, these methodological and clinical discrepancies account for the wide variation in reported prevalence across the literature. Rather than being viewed as competing measures of a single epidemiological parameter, these estimates reflect distinct findings generated under varying operational definitions, sampling frames, patient cohorts, follow-up durations, and modes of symptom ascertainment [24,25,28].

Future epidemiological studies should prioritize standardized case definitions, prospective longitudinal designs, appropriate non-infected control groups, and harmonized outcome assessment, with stratification according to vaccination status, variant, severity of acute infection, age, and other relevant demographic and clinical characteristics [24,25]. Such methodological harmonization will be essential for generating more reliable estimates of the population burden of Long COVID and enabling meaningful comparisons across studies.

## 5. Clinical Phenotypes and Clustering of Long COVID

Given the well-documented diversity within this patient cohort, the clinical manifestation of Long COVID spans a broad spectrum of severity and duration. This variation is underscored by the fact that some individuals sustain persistent symptoms rooted in acute, structurally evident organ damage, whereas others develop new symptoms following a mild infection without clear evidence of tissue injury. This heterogeneity has led researchers to identify distinct or partially overlapping clinical clusters that likely reflect differences in baseline risk factors and underlying pathogenic mechanisms [22,23,32].

To map these variations, major statistical and epidemiological studies have attempted to classify patient cohorts. The Global Burden of Disease Long-COVID study identified three major symptom clusters based on commonly reported manifestations: fatigue with bodily pain or mood swings, respiratory symptoms, and cognitive symptoms, estimating their occurrence at 51.0%, 60.4%, and 35.4% of cases, respectively. Notably, these categories were not mutually exclusive; the overlap was substantial, with approximately 38.4% of individuals presenting with two or all three clusters [26]. Similarly, in a large German population-based cohort (the EPILOC study), Peter et al. demonstrated that fatigue (37.2%) and neurocognitive impairment (31.3%) contributed most significantly to reduced health recovery and working capacity, while musculoskeletal pain further exacerbated occupational limitations. The EPILOC study additionally identified other clinically relevant patterns, including chest symptoms, anxiety/depression, headache/dizziness, and smell or taste disturbances, reinforcing the concept that Long COVID comprises multi-organ patterns with distinct functional consequences [30].

Synthesizing these clustering approaches, including the extensive systematic review by Wang et al., the literature points toward broad, partially overlapping clinical phenotypes [22]. These are predominantly classified into neurological/neurocognitive-dominant, autonomic/fatigue, respiratory, cardiovascular, and systemic presentations [22,33]. However, the exact reproducibility of these clusters across cohorts remains uncertain. As highlighted by Kuodi et al., cluster composition is heavily influenced by the baseline Long COVID definition, the study population, the timing of assessment, the specific symptoms included, and the statistical methods applied [23]. Crucially, as emphasized by Wang et al., statistical clustering identifies patterns of symptom co-occurrence to guide patient management; it does not, by itself, establish discrete biological subtypes or isolated underlying mechanisms [22]. Consequently, clinical phenotyping should be utilized as a foundational framework for future biomarker research. Future studies must determine whether these clinical phenotypes correspond with reproducible molecular, immunological, vascular, or neurological signatures to eventually support precise, individualized treatment strategies [22,33].

## 6. Pathophysiology and Mechanisms of Long COVID

The pathophysiology of Long COVID remains incompletely understood and is unlikely to be explained by a single mechanism. Current evidence points to several interacting biological pathways, including persistent viral or antigenic stimulation, immune dysregulation and neuroinflammation, endothelial and microvascular dysfunction, autonomic abnormalities, metabolic and mitochondrial disturbances, altered gut–brain signaling, and tissue-specific injury [34,35,36,37].

**Figure 1:**
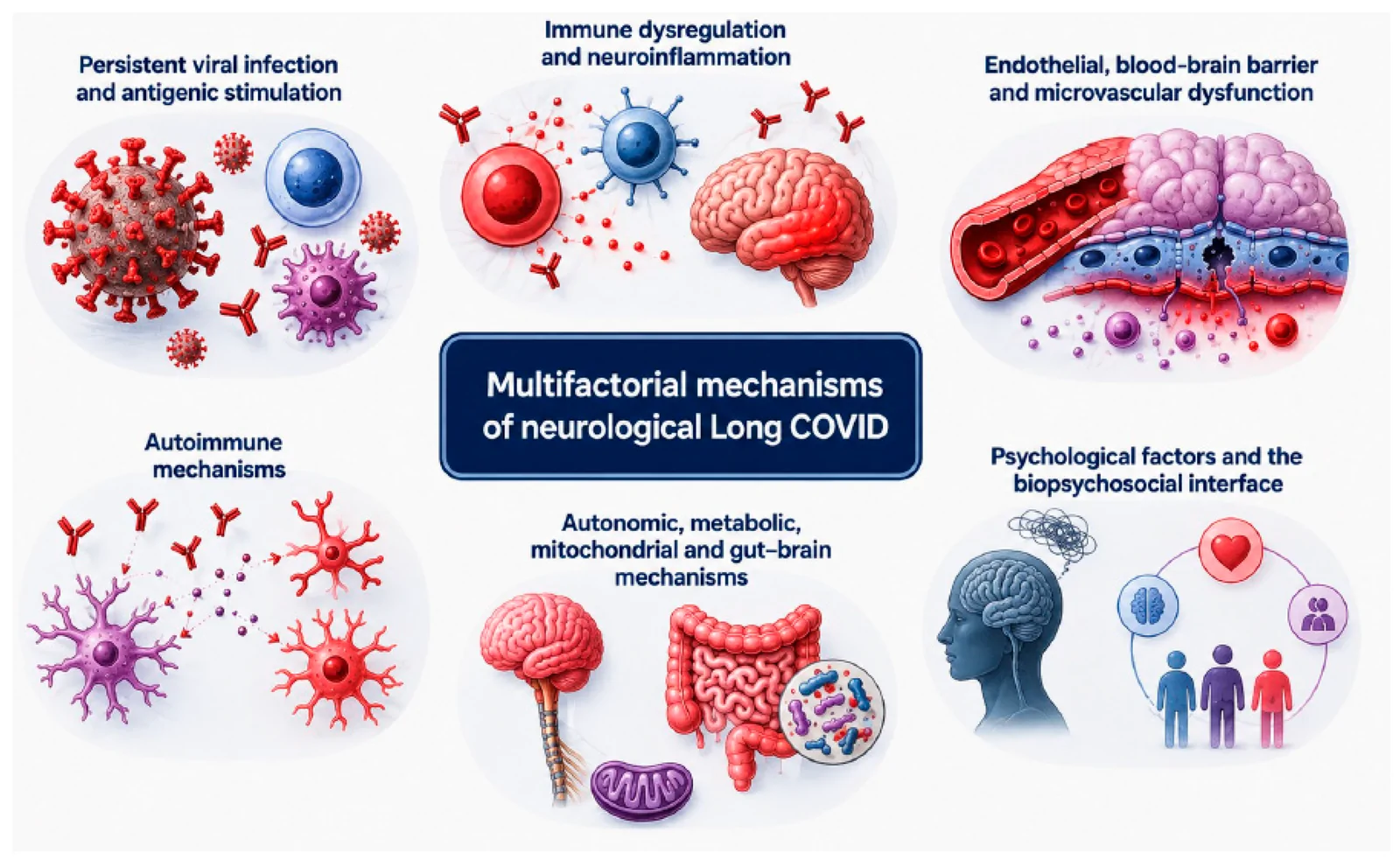
Proposed pathways and candidate mechanisms underlying neurological Long COVID. The figure provides a comprehensive conceptual framework elucidating the intricate candidate mechanisms potentially associated with central nervous system involvement. Ongoing immune activation and inflammatory responses are hypothesized to relate to neuroinflammation, endothelial dysfunction, and blood–brain barrier disruption. Additionally, proposed autoimmune pathways, potential persistence of viral components, and altered gut–brain axis signaling may converge to induce neuronal dysfunction, offering plausible biological explanations for clinical features such as cognitive impairment and fatigue. Together, these proposed pathways help characterize the diverse neurological features observed in Long COVID. This figure was created originally by the authors for this review.

The relative contribution of these mechanisms likely varies across individuals, clinical phenotypes, and disease stages. Rather than operating as mutually exclusive or uniformly expressed processes, these pathophysiological pathways often interact synergistically [14,17,34,35]. The possible pathways underlying neurological Long COVID are synthesized into a conceptual framework in Figure 1.

### 6.1. Persistent Viral Material and Antigenic Stimulation

Persistent viral material or antigenic stimulation has been proposed as one potential mechanism contributing to Long COVID. SARS-CoV-2 RNA or viral proteins have been detected in several tissues after the acute infection, raising the possibility that residual viral components may provide ongoing antigenic stimulation and contribute to persistent immune activation [34,35,36,37].

Detection of viral RNA or proteins does not necessarily indicate ongoing productive viral replication or establish a causal relationship with persistent symptoms. Evidence for viral persistence is heterogeneous across tissues, cohorts, and disease stages. Persistent viral reservoirs are currently best regarded as a plausible mechanism in a subset of patients rather than a universal explanation for Long COVID [36].

### 6.2. Immune Dysregulation and Neuroinflammation

Immune dysregulation and neuroinflammatory processes are among the most extensively investigated mechanisms proposed to contribute to Long COVID. Alterations in cellular and humoral immune responses, together with persistent inflammatory signaling, have been reported in patients with prolonged symptoms [38,39,40,41]. Increased levels of inflammatory mediators, including IL-6 and TNF-α, may contribute to sustained systemic immune activation and, in some patients, activation of neuroimmune pathways [40,41]. Chronic or dysregulated activation of microglia and other components of the neurovascular unit has also been proposed as a mechanism contributing to neurological manifestations such as cognitive dysfunction, fatigue, and headache [14,35]. Inflammatory abnormalities vary substantially between cohorts, and their presence does not by itself establish a causal relationship with individual neurological symptoms.

### 6.3. Endothelial, Blood–Brain Barrier, and Microvascular Dysfunction

Endothelial and microvascular dysfunction may provide an important mechanistic link between systemic immune activation and neurological manifestations. Endothelial activation, altered vascular permeability, disruption of the neurovascular unit, and impaired blood–brain barrier integrity may affect cerebral perfusion and neurovascular coupling, potentially contributing to cognitive dysfunction and other neurological symptoms [14,42]. Alterations in platelet activation, coagulation, and fibrinolytic pathways have also been reported, while fibrin-rich circulating microclots have generated considerable interest as a potential contributor to impaired microcirculation and tissue hypoperfusion [33,42]. The prevalence, specificity, and clinical relevance of these vascular abnormalities remain uncertain, and their direct causal relationship with neurological symptoms has not been consistently established across cohorts.

### 6.4. Autoimmune Mechanisms

Autoimmune mechanisms may contribute to Long COVID in a subset of patients. SARS-CoV-2 infection may induce persistent immune activation and the development of autoantibodies directed against G-protein-coupled receptors and other cellular targets [38,43]. Such immune abnormalities could potentially influence autonomic, vascular, or neurological function [43]. Autoantibodies are not detected uniformly across patients, and their pathogenic significance remains incompletely established. Their presence does not necessarily indicate a single, uniform autoimmune disorder underlying Long COVID.

### 6.5. Autonomic, Metabolic, Mitochondrial, and Gut–Brain Mechanisms

Autonomic, metabolic, mitochondrial, and gut–brain abnormalities may contribute to specific Long COVID phenotypes through interconnected biological pathways. Autonomic dysfunction, including impaired cardiovascular regulation and orthostatic intolerance, has been associated with fatigue, cognitive difficulties, exercise intolerance, and post-exertional symptom exacerbation [5,38,44]. Metabolic and mitochondrial disturbances, including altered cellular energy metabolism and oxidative stress, have also been proposed as contributors to persistent fatigue and reduced exercise or cognitive tolerance [45,46]. Alterations in the gut microbiota and intestinal barrier may influence systemic and neurological function through the gut–brain axis, while changes in tryptophan metabolism and serotonin availability have been proposed as potential links between gastrointestinal, immune, cognitive, and autonomic abnormalities [16,47]. These pathways may interact with inflammatory and vascular processes, although their prevalence, temporal persistence, and contribution to individual neurological phenotypes remain incompletely established.

### 6.6. Psychosocial Factors and the Biopsychosocial Interface

Psychosocial factors may interact bidirectionally with biological mechanisms involved in Long COVID rather than acting as independent contributors [48,49,50]. Persistent symptoms, functional limitations, sleep disturbances, and uncertainty regarding recovery may increase psychological distress and affect coping and daily functioning [48,49,51]. Psychological stress and reduced physical or social activity may also influence autonomic regulation, fatigue, sleep, and symptom perception [49,50]. These interactions do not imply that Long COVID symptoms are primarily psychological or that biological abnormalities can be explained by psychosocial factors [48,49]. A biopsychosocial perspective recognizes that biological, psychological, and social factors may interact to influence symptom severity, persistence, and functional impact [49,50].

### 6.7. Evidence Limitations

The available evidence does not support a single unifying mechanism for Long COVID, and findings across studies are not always consistent [2,3,4]. Differences in patient phenotypes, timing of assessment, severity of the acute infection, vaccination status, prior immunity, circulating viral variants, and methods used to define and measure biological abnormalities may contribute to divergent findings [4,5,24,25,31]. Evidence for persistent viral material, systemic or neuroinflammation, endothelial dysfunction, autoimmunity, and microvascular abnormalities has been reported in some cohorts but has not been consistently demonstrated across all patient populations [14,17,34,35,36,37]. Moreover, biological abnormalities identified at the group level do not necessarily correlate with symptom severity or persist throughout the clinical course [18,20]. These findings suggest substantial heterogeneity between clinical phenotypes and across stages of disease, with proposed mechanisms more likely representing complementary and interacting pathways than mutually exclusive or universally applicable explanations [2,3,17,41].

Current evidence therefore supports a multifactorial model in which persistent viral material or antigenic stimulation, immune dysregulation, neuroinflammation, endothelial and microvascular dysfunction, autonomic abnormalities, and metabolic or mitochondrial disturbances may interact in different combinations across individuals [2,3,41]. Persistent viral material may contribute to sustained immune activation in a subset of patients, with possible downstream effects on endothelial function, microvascular regulation, and blood–brain barrier integrity [14,34,35]. These processes may further interact with neurovascular and CNS immune responses, including neuroinflammatory and glial pathways [14,47]. The relative contribution of these pathways is likely to differ between patients and clinical phenotypes and may change over time [2,3]. Overall, the current evidence is most consistent with a network of interacting mechanisms, while the causal relevance of several proposed pathways remains to be established [3,4].

## 7. Neurological Manifestations

In this review, the term “neurological manifestations” is used broadly to include disorders of the central and peripheral nervous systems, as well as neurocognitive, autonomic, neuromuscular, and persistent chemosensory manifestations following SARS-CoV-2 infection. This approach reflects the heterogeneous neurological spectrum described in the literature and recognizes that some symptoms, including fatigue, post-exertional symptom exacerbation, and myalgia, may have multisystem rather than exclusively neurological mechanisms [2,6,8].

Based on the available literature, we classify the neurological manifestations of Long COVID into seven clinical domains: (1) cognitive and neurobehavioral manifestations, (2) headache and migraine-related disorders, (3) autonomic nervous system dysfunction, (4) peripheral nerve disorders, (5) neuromuscular manifestations, (6) central neurological disorders, and (7) chemosensory manifestations [6,8,9,10]. These domains do not necessarily represent distinct mechanisms or etiological categories. Neurological manifestations may arise in several clinical contexts. Some represent neurological or cognitive consequences of severe acute COVID-19 and its systemic complications, including prolonged respiratory insufficiency, hypoxemia, critical illness, and post-intensive care syndrome [8,13]. Others reflect established organic neurological disease or injury, such as residual deficits following stroke or other cerebrovascular events, inflammatory neurological disorders, or peripheral nerve disorders [6,7]. A further group comprises neurological symptoms for which no established structural or organic correlate can be identified.

Within the latter group, functional neurological disorder (FND; formerly termed conversion disorder) represents a distinct diagnostic entity that should be diagnosed on the basis of positive clinical features rather than by exclusion alone [52]. However, the current literature does not establish FND as a general explanation for neurological symptoms in Long COVID, and available studies have been insufficiently characterized to determine its prevalence or causal relevance in this population [53]. Primary psychiatric disorders, including depressive and anxiety disorders, should likewise be considered separately from neurological sequelae and FND, although clinical overlap may occur [8,54].

### 7.1. Brain Fog

Brain fog is one of the most frequently reported cognitive symptoms in Long COVID [6,55]. The term “brain fog” is not a specific medical diagnosis; it is a descriptive, patient-reported term used to characterize subjective cognitive difficulties—such as impaired concentration, forgetfulness, reduced mental clarity, slowed thinking, and attention deficits [6,55,56]. The exact combination and severity of these symptoms differ significantly among individuals [55,56].

The estimated prevalence of brain fog varies widely depending on the study design. A recent systematic review reported a pooled prevalence of approximately 30% for brain fog among individuals with Long COVID [55]. In a large survey, Perlis et al. found that 40.4% of individuals with Long COVID reported brain fog as a persistent symptom, while 45.7% reported either brain fog or poor memory [29]. Crucially, global findings reveal substantial heterogeneity in these prevalence rates and trajectories across the literature, reflecting differences in study populations, definitions, assessment methods, and follow-up periods [5,24,55].

Subjective cognitive complaints do not necessarily correspond to objectively measurable cognitive impairment on standardized cognitive or neuropsychological testing. Recent systematic reviews indicate that objective cognitive deficits and subjective cognitive symptoms may overlap only partially, with substantial heterogeneity in assessment methods across studies [11,57]. Brain fog is therefore best regarded as a clinical symptom construct rather than a diagnostic equivalent of objectively demonstrated cognitive dysfunction [11,58]. Brain fog has been reported across different age groups and following both mild and severe acute COVID-19 infection [6,55]. Cognitive symptoms may improve over time in some patients, while in others they persist or fluctuate for months or longer [55,56]. Subjective cognitive complaints may also be influenced by fatigue, sleep disturbance, psychological stress, autonomic symptoms, medications, and other systemic factors [15,49,51]. For this reason, brain fog should be interpreted within the broader clinical context and should not, by itself, be considered evidence of a specific neurological lesion.

Currently, no universally accepted diagnostic criteria or standardized clinical recommendations exist for brain fog. When symptoms are persistent or debilitating, objective cognitive assessments are valuable to determine whether subjective complaints are accompanied by measurable deficits and to map the specific cognitive domains involved [11,58].

Regarding potential therapeutic interventions, minor benefits have been reported for a combination of N-acetylcysteine (NAC) and guanfacine [59]. However, current data are strictly restricted to limited clinical observations rather than robust clinical evidence, and randomized controlled trials (RCTs) are entirely lacking. Consequently, these interventions remain strictly investigational and should not be considered established or routinely recommended treatments for Long COVID-related cognitive dysfunction. Adequately powered, controlled studies are urgently needed to determine their actual efficacy and safety profile [6,58].

### 7.2. Cognitive Impairment

Cognitive impairment must be clearly distinguished from subjective brain fog, as it refers to objectively measurable deficits identified through standardized neuropsychological assessments [11,57,58]. This impairment has been documented across various age groups, occurring independently of pre-existing health conditions or the severity of the acute COVID-19 infection [11,60]. Systematic reviews indicate that the most frequently affected domains encompass attention, processing speed, executive functions, and working memory, as well as reasoning, orientation, and verbal learning [11,57]. Depending on the clinical setting, these functions can be evaluated using screening instruments like the Montreal Cognitive Assessment (MoCA), or targeted tasks such as the Trail Making Test, Digit Span, and verbal fluency drills. To address these variations, the IC-CoDi-COVID framework underscores the urgent need for standardized approaches to defining and assessing post-COVID cognitive dysfunction [58].

Objective performance and patient-reported symptoms offer complementary rather than interchangeable data. For instance, a large community-based study utilizing standardized computerized tasks revealed measurable differences in global cognition, primarily affecting memory, reasoning, and executive functions [60]. However, the association between objective test results and self-reported brain fog or memory issues was relatively modest [60]. Consequently, subjective brain fog does not automatically imply an objectively demonstrable deficit, just as true cognitive impairment can occur even when a patient’s subjective complaints are limited [11,57,60].

The clinical course of these cognitive deficits is highly heterogeneous. Symptoms may persist immediately following acute infection, emerge during the post-acute period, or fluctuate over time [11,55,57]. Longitudinal data suggest that cognitive and psychiatric manifestations can remain detectable for prolonged periods, particularly among individuals who required hospitalization or experienced severe acute illness [8,13].

Mechanistically, these disorders are likely driven by a complex interplay of pathways, including neuroinflammation, altered immune signaling, endothelial damage, autonomic dysregulation, and blood–brain barrier abnormalities. Rather than operating in isolation, these mechanisms interact, meaning that cognitive symptoms cannot be universally blamed on direct viral injury to the central nervous system [3,14,47].

Beyond these central processes, experimental and clinical evidence also points to peripheral contributors, including altered gut–brain signaling and disturbances in tryptophan metabolism. A systematic review and meta-analysis found evidence of activation of the tryptophan catabolite and kynurenine pathway in Long COVID, characterized by reduced tryptophan levels and an increased kynurenine-to-tryptophan ratio [16]. Experimental evidence further suggests that SARS-CoV-2 infection may be associated with central cytokine expression, microglial activation, and impaired hippocampal neurogenesis, providing a potential biological basis for persistent cognitive and neuropsychiatric symptoms [61].

Neuroimaging studies have reported structural and functional abnormalities following SARS-CoV-2 infection, including changes in white-matter integrity, regional cerebral blood flow, and cortical measures [62,63]. Although such findings may provide insight into the biological basis of persistent cognitive symptoms, their specificity for Long COVID and relevance at the individual-patient level remain uncertain. Interpretation of neuroimaging findings is further complicated by differences in imaging modalities, acquisition and analytical pipelines, as well as difficulties in distinguishing COVID-19-related changes from pre-existing or nonspecific abnormalities. These neuroimaging abnormalities should therefore be regarded primarily as research findings rather than established diagnostic biomarkers of Long COVID.

Ultimately, cognitive manifestations of Long COVID range from subjective brain fog to objectively measurable cognitive impairment, with only partial overlap between the two [11,57]. Formal objective assessments should be utilized when clinically indicated, particularly when symptoms are persistent, progressive, or functionally disabling, while ensuring that interpretations account for confounding factors such as education, premorbid function, age, fatigue, and mood [6,11,59].

### 7.3. Headache and Migraine-Related Disorders

Headache is one of the most frequently reported neurological manifestations following SARS-CoV-2 infection [6,64]. For some patients, it manifests as an exacerbation of a pre-existing primary headache disorder, such as migraine, whereas in others the infection may trigger a new headache phenotype, including new daily persistent headache or a migraine-like disorder [64,65,66].

A meta-analysis by Fernández-de-las-Peñas et al. demonstrated that post-COVID headache may persist beyond the acute phase, with estimated prevalence rates of 10.2% at 30 days, 16.5% at 60 days, 10.6% at 90 days, and 8.4% after six months [66]. Long-term neurological studies have likewise identified headache and migraine among persistent neurological manifestations reported during follow-up [7,67].

Proposed mechanisms include persistent immune activation and cytokine-mediated inflammation, which may contribute to sustained activation of the trigonavascular system. Endothelial and vascular dysfunction may further contribute to headache persistence, while alterations in serotonin and tryptophan metabolism have also been proposed as potential contributors [16,64,67]. These mechanisms remain incompletely established and are likely to interact rather than represent independent pathways.

Persistent headache may occur following both mild and severe acute infection [6,66]. Clinical assessment should nevertheless distinguish persistent post-COVID headache from secondary headache caused by cerebrovascular, inflammatory, or other structural complications [6,64].

### 7.4. Sensory Manifestations and Peripheral Nerve Disorders

Sensory manifestations are common and heterogeneous features of post-COVID-19 neurological syndromes. Patients may report a range of dysesthetic and neuropathic symptoms, including burning pain, tingling, paresthesias, numbness, and electric-shock-like sensations [6,68]. Symptoms may be predominantly distal and symmetric, particularly affecting the hands and feet. In a subset of patients, the clinical phenotype may be compatible with small-fiber neuropathy (SFN) or related peripheral nerve dysfunction [69,70,71]. However, the pathophysiological relationship between SFN and Long COVID remains incompletely established, and sensory symptoms alone should not be considered diagnostic of SFN.

Beyond small-fiber involvement, persistent sensory or motor deficits may occasionally reflect a defined peripheral neurological disorder, including Guillain–Barré syndrome (GBS), inflammatory demyelinating neuropathies, or other polyneuropathic phenotypes reported after SARS-CoV-2 infection [68,72,73]. A recent systematic review and meta-analysis including more than 15.8 million COVID-19 cases found that neuromuscular conditions and related symptoms remain relevant during long-term follow-up, although their prevalence and trajectories vary considerably between individual conditions [73]. GBS has also been specifically reported following SARS-CoV-2 infection, with a systematic review describing a broad range of clinical and electrophysiological presentations [72]. These conditions should be distinguished from nonspecific paresthesias associated with Long COVID because they require appropriate neurological assessment and, when clinically indicated, electrophysiological evaluation and disorder-specific management [6,68].

The underlying mechanisms of sensory symptoms in Long COVID remain incompletely understood, and clinical trajectories may range from gradual improvement to persistent or fluctuating symptoms [68,73]. Accordingly, chronic sensory manifestations may reflect peripheral, central, or multisystem processes depending on the clinical context. Diagnostic evaluation should be guided by the clinical phenotype and neurological examination. When a peripheral neuropathy is suspected, nerve conduction studies and electromyography may help identify large-fiber involvement, whereas additional investigations, including skin biopsy for intraepidermal nerve fiber density or autonomic and small-fiber testing, may be considered when SFN is clinically suspected despite normal routine electrophysiological studies [69,70,71]. Neuroimaging should be reserved for patients whose clinical presentation raises suspicion of a central neurological disorder.

### 7.5. Autonomic Dysfunction

Autonomic dysfunction is an important component of the neurological phenotype of Long COVID. Common manifestations include orthostatic intolerance, postural orthostatic tachycardia syndrome (POTS), abnormal sweating, flushing, and orthostatic dizziness, while orthostatic hypotension (OH) and neurocardiogenic syncope (NCS) have also been reported [15,38,74,75]. Other symptoms that may reflect autonomic involvement include palpitations, gastrointestinal and genitourinary disturbances, altered sweating, dry eyes or mouth, and changes in vascular regulation [15,38,76].

The frequency and severity of autonomic symptoms vary between studies, partly because of differences in patient selection, diagnostic criteria, timing of assessment, and methods used to evaluate autonomic function [15,76]. Longitudinal observations indicate that symptoms may fluctuate over time, with some patients experiencing partial improvement followed by recurrence or persistence during longer-term follow-up [38,76]. A recent systematic review and meta-analysis further support the presence of persistent neurocardiac autonomic abnormalities in patients with post-COVID-19 condition, although substantial heterogeneity remains across studies [77].

Several mechanisms have been proposed to contribute to autonomic dysfunction after SARS-CoV-2 infection, including autonomic dysregulation, endothelial dysfunction, immune-mediated mechanisms, and small-fiber involvement [15,38,74,78]. Small-fiber dysfunction is of particular interest because small autonomic fibers contribute to cardiovascular, sudomotor, gastrointestinal, and genitourinary regulation. However, autonomic symptoms alone do not establish a diagnosis of small-fiber neuropathy, and the relationship between SFN, dysautonomia, and Long COVID remains under investigation [69,75]. A recent review further emphasizes the overlapping neuroimmune, autonomic, mitochondrial, and cerebral hypoperfusion mechanisms proposed across POTS, ME/CFS, and Long COVID, although these mechanisms remain incompletely established [78].

Autonomic dysfunction should therefore be regarded as a heterogeneous clinical manifestation rather than a single monolithic autonomic disorder. When clinically indicated, assessment may include orthostatic vital signs, active stand testing, autonomic testing, or other targeted investigations according to the predominant symptoms and suspected syndrome [15,74,76]. Objective assessment is particularly important when POTS, OH, NCS, or another defined autonomic disorder is suspected, as these conditions require appropriate diagnostic criteria and disorder-specific management.

### 7.6. Myalgia and Musculoskeletal Manifestations

Myalgia and arthralgia are among the persistent musculoskeletal symptoms reported following SARS-CoV-2 infection [8,79]. Patients may describe diffuse or regional muscle and joint pain, often with a fluctuating or relapsing–remitting course [79]. Musculoskeletal complaints have been reported in both non-hospitalized and hospitalized populations, although estimates vary considerably between cohorts [28,79,80]. Differences in disease severity, hospitalization status, duration of follow-up, patient characteristics, and methods of symptom ascertainment may contribute to this variability.

In hospitalized patients, persistent pain may also be influenced by factors related to severe acute illness and intensive care, including prolonged immobilization, muscle deconditioning, mechanical ventilation, critical illness-related weakness, and positioning during hospitalization [8,81]. These factors warrant careful consideration when evaluating musculoskeletal symptoms following severe acute COVID-19 illness, given their potential to contribute independently of Long COVID-specific pathways.

Several mechanisms have been proposed for post-COVID myalgia, including systemic inflammatory and immune-mediated responses, altered muscle metabolism, endothelial or microvascular dysfunction, and physical deconditioning [80,81]. The mechanisms underlying persistent myalgia, however, remain incompletely defined. Prolonged musculoskeletal symptoms should therefore not be attributed solely to persistent systemic inflammation or sustained cytokine dysregulation [79,80].

Clinical assessment should consider alternative or coexisting causes of persistent myalgia, including medication effects, rheumatological disease, endocrine or metabolic disorders, sleep disturbance, physical deconditioning, and neurological conditions. Management should be based on the underlying clinical phenotype and functional impact rather than on an assumed single mechanism.

### 7.7. Central Neurological Disorders

Objectively defined central neurological disorders have also been reported following SARS-CoV-2 infection, including encephalopathy, cerebrovascular disease, seizures, movement disorders, and, less commonly, inflammatory neurological disorders. A large population-based cohort study by Xu et al. demonstrated increased risks of several neurological outcomes during the post-acute period, including ischemic and hemorrhagic stroke, cognition and memory disorders, seizures, extrapyramidal and movement disorders, and encephalitis or encephalopathy [82]. The systematic review by Giussani et al. likewise identified a broad range of neurological outcomes during follow-up, although their frequency and clinical significance varied considerably between studies [7].

It is important to distinguish these specific clinical entities from the nonspecific neurological complaints commonly reported in Long COVID [6,8,82]. Encephalopathy, for example, requires a documented alteration in mental status established by clinical assessment, whereas cerebrovascular disease is diagnosed on the basis of neurological findings and appropriate neuroimaging. Similarly, seizures require a compatible clinical presentation supported, when appropriate, by electroencephalographic evaluation, while movement disorders and inflammatory neurological conditions require appropriate neurological assessment and condition-specific diagnostic investigations.

The pathophysiological mechanisms underlying these disorders are complex and heterogeneous. Proposed pathways include systemic inflammation, endothelial and vascular dysfunction, thromboinflammatory processes, immune-mediated injury, hypoxia, and alterations in neuroimmune signaling [14,47,83]. Increasing evidence suggests that interactions between peripheral immune responses and the central nervous system may contribute to neurological dysfunction in Long COVID [83]. However, the available evidence does not support the view that neurological disorders occurring after COVID-19 can be uniformly attributed to direct viral invasion of the central nervous system [3,47].

When a specific neurological disorder is identified, its diagnosis, treatment, prognosis, and follow-up should primarily follow the established clinical guidelines for that condition. Such disorders should therefore not be attributed nonspecifically to Long COVID without appropriate clinical and diagnostic evaluation [6,82].

### 7.8. Chemosensory Manifestations

Anosmia and hyposmia are distinctive manifestations associated with SARS-CoV-2 infection [84,85]. Although they commonly develop during the acute phase, olfactory dysfunction may persist into the post-acute period [85].

In the systematic review by Giussani et al., anosmia/hyposmia was among the neurological manifestations reported during follow-up, although its prevalence generally declined over time [7].

Persistent chemosensory dysfunction may occur independently or together with cognitive symptoms and headache [85,86]. Its pathophysiology is likely multifactorial and may involve local epithelial injury, inflammatory responses, altered olfactory pathways, and, in some cases, central nervous system mechanisms [84,85].

## 8. Emerging Biomarkers and Future Perspectives in Neurological Long COVID

**Figure 2:**
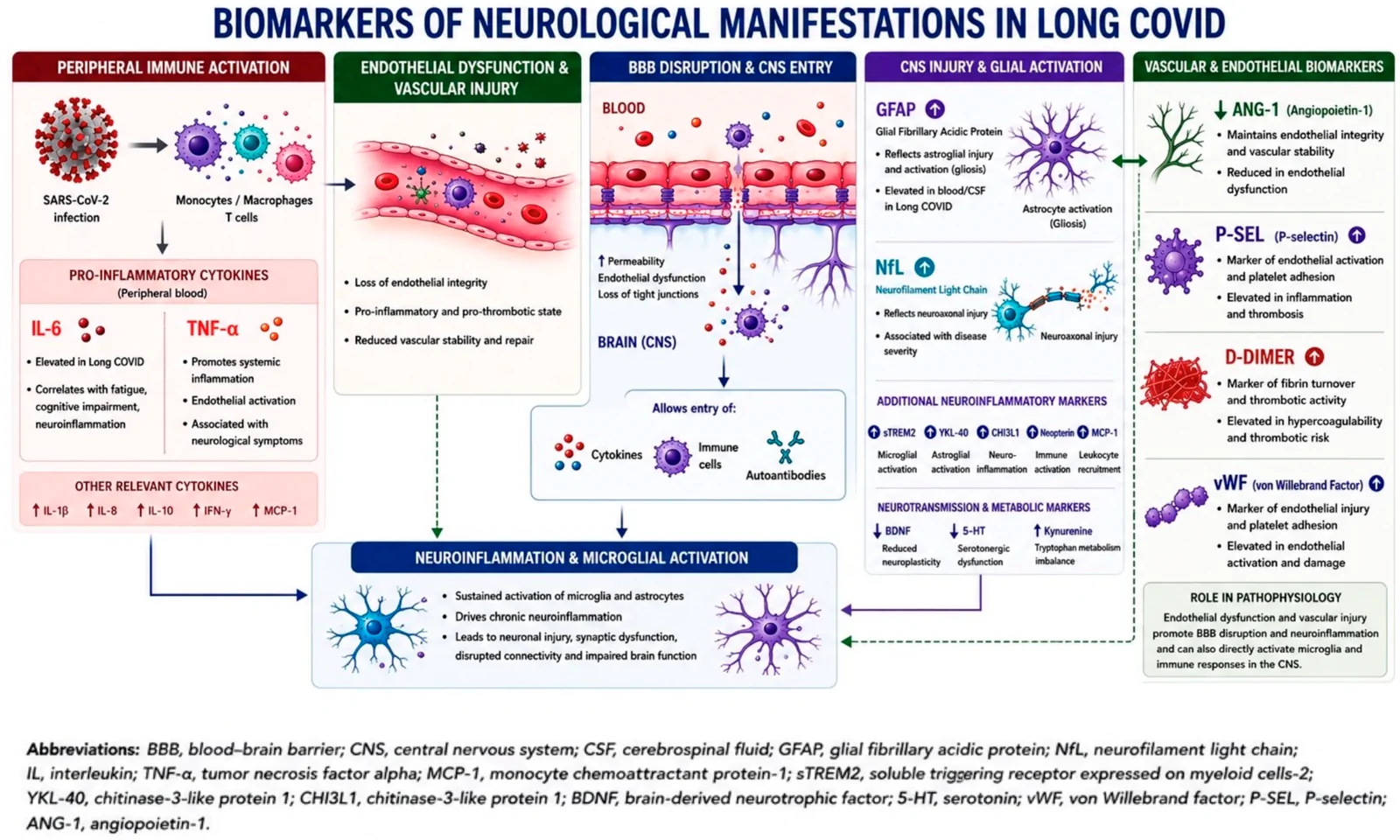
Proposed biological pathways and candidate biomarkers involved in neurological Long COVID. These neurological conditions are hypothesized to be associated with interacting processes like immune dysregulation, neuroinflammation, and blood–brain barrier disruption. The figure outlines the specific candidate biomarkers evaluated in this analysis, including reported associations with inflammatory cytokines, vascular, endothelial, astroglial, and neuroaxonal damage markers, alongside immune-related changes. These candidate biomarkers may clarify disease heterogeneity and support future patient stratification frameworks. This figure was created originally by the authors for this review.

Growing evidence indicates that neurological manifestations of Long COVID may involve several interacting processes, including immune dysregulation, neuroinflammation, endothelial and microvascular dysfunction, autonomic abnormalities, and neuronal or glial injury. This biological complexity makes it unlikely that a single biomarker will capture the full clinical spectrum of neurological Long COVID. Current research is therefore increasingly focused on multimarker profiles that may be associated with specific clinical phenotypes and underlying biological pathways [18,20,21,87]. The conceptual framework presented in Figure 2 integrates candidate biomarkers within the broader biological pathways implicated in neurological Long COVID. Rather than presenting biomarkers as isolated abnormalities, the figure illustrates their potential relationships with neuroinflammation, neuronal and astroglial injury, endothelial and microvascular dysfunction, blood–brain barrier disruption, and immune dysregulation [14,18,19,20,88]. It also emphasizes the potential value of combining biomarker findings with clinical phenotyping, while recognizing that these proposed relationships require further clinical validation.

When evaluating these biological indices, a critical distinction must be maintained between generic, systemic biomarkers associated with Long COVID as a whole, and specific biomarkers explicitly validated in cohorts with defined neurological phenotypes. Markers such as generic inflammatory cytokines or global vascular markers often reflect broad, systemic post-viral processes. Conversely, markers tied to direct central nervous system pathways are required to map distinct neurological sub-phenotypes [19,20,21].

Candidate biomarkers investigated in neurological Long COVID encompass several levels of disease biology. NfL has been studied as a marker of axonal or neuronal injury, whereas GFAP may reflect astroglial activation and, in some settings, abnormalities involving the neurovascular unit or blood–brain barrier [14,19,89]. Inflammatory mediators, including IL-6 and TNF-α, have been investigated as indicators of systemic or neuroimmune activation [42,87]. Vascular and endothelial markers, including von Willebrand factor (vWF), P-selectin, angiopoietin-1 (Ang-1), D-dimer, and adhesion molecules, have also been examined in relation to endothelial activation, thrombo-inflammatory processes, and microvascular alterations [90,91]. Autoantibodies, including antibodies directed against G-protein-coupled receptors, have additionally been investigated in patients with autonomic manifestations [44,92]. Most of these markers are biologically informative but are not disease-specific, and their interpretation depends on the clinical phenotype and the context in which they are measured.

Recent proteomic studies have further supported a multimarker rather than single-marker approach. Instead of identifying one molecule that consistently distinguishes all patients with Long COVID, these studies suggest that combinations of inflammatory, immune, vascular, and tissue-injury markers may be associated with particular clinical phenotypes [20,21]. Such findings are consistent with the concept that neurological Long COVID comprises biologically heterogeneous patient subgroups rather than a single molecular entity [20,22].

The potential clinical value of a biomarker depends on its intended application. While diagnostic tools should accurately differentiate Long COVID from appropriate comparison cohorts, prognostic indicators are required to map disease trajectories, persistence, and therapeutic responses. Currently, most investigated candidates serve primarily as phenotype-associated or mechanistic markers, helping characterize biological pathways rather than serving as validated clinical instruments [18,20,21].

One recent study illustrates both the potential and the current validation challenges of this approach. A combination of C5a, TGF-β1, and gliomedin distinguished patients with neurological Long COVID from control groups, with a reported sensitivity of 94% and specificity of 86% [21]. However, it is essential to contextualize these parameters methodologically: the panel was evaluated in a relatively modest discovery cohort consisting of 48 non-hospitalized Neuro-PASC patients compared against 20 convalescent controls and 24 healthy individuals [21]. Because the study lacks reported confidence intervals for these performance metrics and utilizes an internal discovery-based screening approach without a large independent replication cohort, there remains a notable risk of statistical overfitting [21]. Consequently, while these findings are encouraging, this specific biomarker combination requires rigorous external validation across heterogeneous cohorts before its clinical maturity and diagnostic readiness can be established [21].

Candidate markers instead show significant inconsistency across various cohorts. Controlled studies have reported no significant differences in plasma NfL or GFAP between some individuals with Long COVID and recovered controls [19,93]. Recent evidence from a 2026 case–control study of individuals with persistent neurological symptoms further highlights the potential relevance of inflammatory and neuroinjury blood biomarkers in neurological Long COVID, while also demonstrating differences according to the severity of the initial infection [94]. Differences in patient phenotypes, disease duration, control populations, sampling time, assay methodology, and the biological processes captured by individual markers may contribute to these discrepancies [2,4,41,87]. They also highlight the distinction between markers of general neuronal or astroglial injury and biomarkers with sufficient specificity to identify Long COVID or a particular neurological phenotype.

Further progress from promising candidates to clinically useful biomarkers will require standardized sampling and analytical procedures, longitudinal assessment, and independent validation. Relevant methodological factors include sample type, timing of collection, processing and storage, assay platform, calibration, and reporting of analytical performance [4,18,87]. Candidate biomarkers should be evaluated in adequately powered longitudinal cohorts and tested against appropriate comparison groups, including healthy individuals, individuals who recovered from COVID-19 without persistent symptoms, and patients with clinically similar non-COVID conditions.

Integration of biomarkers with standardized clinical phenotyping may be particularly informative. Rather than seeking a universal biomarker for Long COVID, future studies could evaluate phenotype-specific biological signatures associated with cognitive dysfunction, autonomic dysfunction, fatigue/post-exertional symptom exacerbation, headache, sleep disturbance, or neurological manifestations [20,21,22]. Multimodal panels incorporating markers of neuronal injury (NfL), astroglial activation (GFAP), immune activation (e.g., IL-6 and TNF-α), endothelial and vascular dysfunction (e.g., vWF, P-selectin, Ang-1, D-dimer), and phenotype-specific immune markers may provide a more informative biological profile than any individual marker [18,19,20,21,87,90,91,92,93].

Neuroimaging may provide an additional layer of biological characterization. Structural and functional studies have reported alterations in cortical measures, white-matter integrity, cerebral perfusion, and regional brain metabolism following SARS-CoV-2 infection [62,63]. These findings remain heterogeneous and are generally derived from group-level comparisons rather than abnormalities validated for individual-patient diagnosis [62,63]. Neuroimaging is currently more useful for investigating disease biology and supporting biological phenotyping than as a specific diagnostic biomarker.

The future direction of biomarker research is likely to lie in integrated biological profiling rather than the identification of a single universal marker. An important objective will be to determine whether reproducible combinations of molecular, vascular, neuronal, and immune markers, interpreted alongside standardized clinical phenotypes and longitudinal trajectories, can improve patient stratification, prognostic assessment, or treatment selection [2,4,20,21,22]. At present, such multimarker signatures should be regarded as promising research frameworks rather than established clinical tools.

## 9. Long COVID Management

### 9.1. Testing and Diagnosis

The diagnosis of Long COVID remains primarily clinical, based on a comprehensive assessment of persistent, recurrent, or newly emerging symptoms following SARS-CoV-2 infection. No single laboratory test, biomarker, or imaging modality currently has sufficient sensitivity and specificity to establish the diagnosis in routine clinical practice [1,95,96]. Diagnostic assessment should therefore integrate the clinical history, temporal relationship between SARS-CoV-2 infection and subsequent symptoms, predominant symptom phenotype, functional limitations, and relevant physical, neurological, and cognitive findings.

The WHO clinical definition of post-COVID-19 condition provides an important operational framework for clinical and research purposes, but its application to individual patients may remain challenging because of the heterogeneous and fluctuating nature of the condition [1,5]. Phenotype-based characterization can complement this framework by identifying the predominant clinical domain, such as cognitive, neurological, autonomic, cardiopulmonary, fatigue/PESE, or multisystem manifestations [2,22,23].

Characterization of symptom duration and trajectory, severity of the initial infection, vaccination and reinfection history, and relevant comorbidities may further assist clinical assessment and facilitate comparison between research cohorts. This type of stratification should be considered a pragmatic interim approach rather than a validated diagnostic classification [2,5].

Clinical evaluation should also actively consider alternative or coexisting diagnoses, particularly when symptoms are severe, progressive, atypical, or associated with objective neurological or systemic abnormalities. Depending on the presenting phenotype, investigations may include targeted laboratory testing, cardiopulmonary assessment, neurological or neuropsychological evaluation, autonomic testing, neuroimaging, electrophysiological studies, or other investigations when clinically indicated [6,95,96]. Testing should be guided by the presenting symptoms and examination findings rather than performed as an extensive routine diagnostic panel in all patients.

The absence of an objective abnormality on routine clinical tests cannot rule out Long COVID, particularly in patients with predominantly subjective or fluctuating symptoms. At the same time, persistent symptoms should not automatically be attributed to Long COVID until other potentially treatable diseases are ruled out. This distinction is especially relevant to neurological manifestations [6,95].

For patients reporting cognitive difficulties, subjective brain fog should be distinguished from objectively measurable cognitive impairment; standardized cognitive screening or comprehensive neuropsychological assessment may be appropriate when symptoms are persistent, functionally significant, or diagnostically uncertain [6,11,58]. Similarly, autonomic symptoms may warrant orthostatic assessment or formal autonomic testing when clinically indicated, while persistent sensory or motor symptoms may require neurological examination, electrophysiological studies, small-fiber assessment, or neuroimaging according to the suspected underlying disorder [6,15,69,76].

The interpretation of diagnostic findings should also take into account the distinction between symptoms without an established structural correlate and objectively defined neurological disease.

When identifying a specific neurological disorder, such as stroke, inflammatory myelitis, Guillain–Barré syndrome, or another established peripheral or central neurological condition, clinicians should manage the case according to classic neurological practices rather than attributing all findings nonspecifically to Long COVID [6,72,82]. Conversely, normal routine investigations do not necessarily invalidate clinically significant symptoms or functional impairment.

Longitudinal clinical assessment is therefore particularly important, as symptoms may fluctuate, resolve, recur, or evolve over time. Reassessment should focus on symptom trajectory, functional impact, emerging objective findings, and the possibility of alternative or coexisting diagnoses rather than relying on a single cross-sectional assessment [5,23,95].

### 9.2. Treatment Options

The management of Long COVID remains primarily individualized and symptom-directed, reflecting the substantial clinical heterogeneity of the condition and the absence of an established disease-modifying therapy. Current clinical guidance emphasizes a patient-centered approach focused on the symptoms and functional limitations that are most burdensome to the individual, with treatment goals established through shared decision-making and regular reassessment [95,97]. Management should therefore be tailored to the predominant clinical phenotype, functional capacity, comorbidities, safety considerations, and treatment tolerance rather than based on a uniform therapeutic protocol [6,95]. To ensure clinical safety and transparency, therapeutic strategies must be clearly categorized based on the strength of their underlying evidence.

#### 9.2.1. Established Guideline Recommendations

Established clinical approaches from major health organizations, such as the U.S. Centers for Disease Control and Prevention (CDC) and the World Health Organization (WHO), emphasize patient-centered, symptom-specific management [95,97]. These established strategies are directed toward the patient’s most burdensome symptoms and functional limitations, alongside the appropriate evaluation and treatment of underlying or coexisting medical conditions.

Pharmacological treatment also falls under established guidelines and remains predominantly symptom-directed. Individual symptoms and newly identified medical conditions should be managed according to established clinical indications, taking into account comorbidities, potential drug interactions, risk–benefit considerations, and treatment tolerance [6,95,96]. For specific neurological manifestations, treatment should be guided by the clinical phenotype and by established approaches used for the corresponding neurological condition [6,95].

Within this established framework, before initiating any physical exercise-based rehabilitation, clinicians must strictly assess for post-exertional symptom exacerbation (PESE), exertional desaturation, cardiac impairment, and other conditions that may contribute to reduced exercise tolerance [95,96]. In patients without PESE and without relevant cardiopulmonary contraindications, symptom-titrated physical activity or exercise-based rehabilitation may be offered, individualized strictly according to clinical response [95]. In patients experiencing PESE, rehabilitation should instead prioritize pacing and energy conservation, rather than fixed or graded increases in exercise intensity that may exacerbate symptoms.

#### 9.2.2. Conditional Recommendations Based on Low-Certainty Evidence

Several widely utilized supportive and rehabilitative interventions currently rely on conditional recommendations supported by low or very low-certainty evidence [95]. For patients experiencing PESE, the WHO conditionally recommends education and skills training in energy conservation and pacing, with activity and rest adjusted flexibly according to symptoms and individual energy limits [95]. Importantly, fixed incremental increases in physical activity or graded exercise should not be offered to patients experiencing PESE, and these recommendations must be interpreted in the context of clinical judgment and patient preferences.

Similarly, cognitive rehabilitation and other supportive strategies, such as education, compensatory strategies, and individualized cognitive exercises, are conditionally recommended when persistent cognitive symptoms interfere with everyday functioning [95]. These supportive interventions must be customized according to the patient’s cognitive profile while accounting for overlapping factors such as fatigue, sleep disturbance, mood, and autonomic symptoms [6,95]. For autonomic manifestations, management strategies such as fluid and salt optimization, compression measures, and individualized rehabilitation approaches have been described, although the available evidence remains heterogeneous and does not support a standardized treatment protocol [76,98].

#### 9.2.3. Investigational Interventions

At present, no disease-modifying pharmacological therapy has been established as a standard treatment for Long COVID, and several proposed pharmacological and non-pharmacological interventions remain strictly investigational [2,3,6].

Neuromodulatory approaches, including repetitive transcranial magnetic stimulation (rTMS) and intermittent theta-burst stimulation (iTBS), fall into this experimental category. While completed pilot studies suggest potential benefits in selected patients for managing cognitive dysfunction, depression, fatigue, and insomnia, the available evidence remains severely limited by small sample sizes, heterogeneous treatment protocols, and short follow-up periods [99,100,101]. Accordingly, rTMS and iTBS must currently be regarded as investigational interventions rather than established treatments, and their clinical role requires definitive confirmation in adequately powered randomized controlled trials.

Importantly, the evidence base for TMS in Long COVID remains under active development. A 2025 systematic review and meta-analysis protocol was published to evaluate the therapeutic potential of repetitive transcranial magnetic stimulation in Long COVID, underscoring that the field remains investigational and that a definitive synthesis of efficacy evidence was still pending [102]. Therefore, the existence of ongoing systematic evaluation should not be interpreted as evidence that TMS has already demonstrated clinical efficacy.

Overall, treatment of Long COVID should remain individualized and responsive to the patient’s dominant clinical phenotype, with established symptomatic and rehabilitative approaches prioritized according to current guidelines, conditional interventions applied selectively according to symptom profile and evidence certainty, and experimental therapies reserved for appropriately selected clinical or research settings [6,95,96].

### 9.3. Recovery from Long COVID

Recovery from Long COVID is highly heterogeneous, and the duration and trajectory of neurological manifestations vary substantially between individuals. Although many patients experience gradual improvement during the first months following acute infection, long-term follow-up studies extending beyond two years continue to demonstrate persistent neurological symptoms in some patients, including cognitive impairment, fatigue, headache, autonomic dysfunction, and other neurological manifestations [7,103,104,105].

Prospective cohort studies have reported that persistent cognitive and psychiatric symptoms can remain detectable up to 2–3 years specifically among individuals who required hospitalization for COVID-19 [103]. Concurrently, large-scale population-based research demonstrates that recovery from severe fatigue and cognitive difficulties is frequently incomplete over prolonged timelines [105]. Furthermore, population-based follow-up data from the second year after acute infection confirm that persistent symptoms and functional abnormalities may remain detectable well beyond the initial post-acute period [103,106].

The clinical trajectory may be fluctuating rather than continuously progressive or steadily improving. Symptoms may resolve, recur, or vary in severity over time, and different neurological manifestations may follow different recovery trajectories within the same individual [7,76]. Persistent symptoms at a given time point should not necessarily be interpreted as evidence of irreversible neurological damage, nor should prolonged symptoms be assumed to reflect a uniform, ongoing process of recovery. Long-term follow-up should be guided by the individual clinical phenotype, functional impact, and evolution of symptoms.

Long COVID may follow heterogeneous longitudinal trajectories, with some patients experiencing gradual improvement or recovery, while others develop persistent, fluctuating, or delayed worsening of symptoms [7,23]. In the prospectively followed RECOVER-Adult cohort, eight distinct longitudinal symptom profiles were identified over 3–15 months after infection, including persistent, intermittently high, improving, and delayed-increasing symptom trajectories, further supporting substantial heterogeneity in the course of Long COVID [107].

Persistent symptoms may also remain detectable several years after SARS-CoV-2 infection. A 2025 systematic review and meta-analysis found that approximately 20% of COVID-19 survivors had at least one unresolved symptom three years after infection, although the pooled estimate encompassed a broad range of persistent post-COVID manifestations rather than neurological symptoms specifically [108].

Persistent neurological deficits should also not always be interpreted as an ongoing manifestation of Long COVID. When SARS-CoV-2 infection is complicated by a defined neurological disorder, such as ischemic or hemorrhagic stroke, Guillain–Barré syndrome, established peripheral neuropathy, or inflammatory myelitis, resulting deficits may persist independently of resolution of the systemic manifestations of COVID-19. Once established, these conditions should be regarded as distinct neurological disorders rather than indefinitely attributed to an ongoing post-viral recovery process. Their prognosis, rehabilitation, follow-up, and treatment should be guided primarily by the specific neurological diagnosis and established clinical guidelines, as for comparable disorders arising from non-COVID etiologies.

The course of neurological Long COVID ranges from gradual symptom resolution to prolonged or persistent manifestations. Clinical phenotype, severity of the initial illness, comorbidities, and functional consequences may all influence recovery trajectories [2,7,23,104]. Recognizing this variability is important for realistic prognostic counseling, appropriate long-term follow-up, and individualized management, while avoiding the assumption that all persistent neurological deficits represent an ongoing process of recovery from Long COVID.

## 10. Conclusions

Long COVID represents a heterogeneous and multifactorial condition in which persistent neurological, cognitive, autonomic, peripheral, neuromuscular, and chemosensory manifestations may arise through distinct and potentially overlapping mechanisms [2,4,14]. The evidence reviewed in this manuscript indicates that these manifestations should not be attributed to a single pathophysiological process. Primary organic neurological involvement may reflect neuroinflammation, endothelial and blood–brain barrier dysfunction, immune-mediated mechanisms, and neuronal or glial injury, whereas neurological and cognitive manifestations may also arise secondarily from systemic factors such as hypoxia, respiratory insufficiency, systemic inflammation, and prolonged critical illness [2,4]. Primary psychiatric disorders and functional neurological disorders represent additional clinically important but mechanistically distinct components of the post-COVID spectrum [6,21].

Despite substantial advances in understanding the biological basis of Long COVID, important uncertainties remain regarding the relative contribution, temporal evolution, and clinical significance of individual mechanisms. No single biomarker or biomarker panel has yet demonstrated sufficient specificity, reproducibility, and clinical validity to support routine diagnosis or prognostic stratification of neurological Long COVID [19,21]. Likewise, no disease-modifying therapy has yet been validated, and current management remains primarily individualized, symptom-directed, and phenotype-oriented [95,96].

Three priorities should guide future research. First, standardized neurological phenotyping and harmonized outcome measures are needed to improve comparability between studies and determine whether currently identified clinical clusters represent reproducible phenotypes or patterns of symptom co-occurrence [22]. Second, candidate biomarkers require longitudinal and independent validation in well-characterized cohorts, with particular emphasis on multimodal biomarker signatures associated with specific neurological phenotypes [19,21]. Third, phenotype-stratified clinical trials are needed to determine whether targeted therapeutic strategies can improve outcomes in clinically and biologically defined patient subgroups [102].

Addressing these priorities may facilitate the transition from descriptive characterization of neurological Long COVID toward validated biomarkers, evidence-based therapeutic strategies, and more precise, patient-centered care.

## Data Availability

No new data were created or analyzed in this study. Data sharing is not applicable to this article.

## Supplementary Materials

The following supporting information can be downloaded at https://www.mdpi.com/article/10.3390/jcm15187163/s1.
