# Supplementary material for: Neurological Manifestations of Long COVID: Current Evidence, Emerging Concepts, and Future Perspectives
Source: J Clin Med. 2026 Sep 15;15(18):7163. doi: 10.3390/jcm15187163 (PMC13607159; doi:10.3390/jcm15187163)
Supplement: Supplementary file 1 [file jcm-15-07163-s001.zip › jcm-4488110-supplementary.pdf]

Supplementary Table S1.

Database / Platform	Search Date	Exact Search Strings / Keywords Used	Purpose / Target
PubMed	July 15, 2026	("Post-Acute COVID-19 Syndrome"[Mesh] OR "Long COVID"[tiab] OR "post-COVID-19 condition"[tiab]) AND ("neurologic manifestations"[tiab] OR "neurological manifestations"[tiab] OR "neurologic symptoms"[tiab] OR "neurological symptoms"[tiab])	Primary core literature identification
PubMed	July 15, 2026	("Post-Acute COVID-19 Syndrome"[Mesh] OR "Long COVID"[tiab] OR "post-acute sequelae of COVID-19"[tiab]) AND ("biomarkers"[tiab] OR "neuroinflammation"[tiab] OR "blood-brain barrier"[tiab])	Targeted mechanistic and biomarker search
Google Scholar	July 15, 2026	"Long COVID" neurological biomarkers treatment	Cross-referencing and screening for potentially missed records
Google Search	July 15, 2026	Long COVID neurological management guidelines WHO CDC	Restricted exclusively to official institutional guidelines and consensus documents
